# Mucosal and Cutaneous Human Papillomaviruses Detected in Raw Sewages

**DOI:** 10.1371/journal.pone.0052391

**Published:** 2013-01-14

**Authors:** Giuseppina La Rosa, Marta Fratini, Luisa Accardi, Graziana D'Oro, Simonetta Della Libera, Michele Muscillo, Paola Di Bonito

**Affiliations:** 1 Department of Environment and Primary Prevention, Istituto Superiore di Sanità, Rome, Italy; 2 Department of Infectious Parasitic and Immune-mediated Diseases, Istituto Superiore di Sanità, Rome, Italy; Columbia University, United States of America

## Abstract

Epitheliotropic viruses can find their way into sewage. The aim of the present study was to investigate the occurrence, distribution, and genetic diversity of Human Papillomaviruses (HPVs) in urban wastewaters. Sewage samples were collected from treatment plants distributed throughout Italy. The DNA extracted from these samples was analyzed by PCR using five PV-specific sets of primers targeting the L1 (GP5/GP6, MY09/MY11, FAP59/64, SKF/SKR) and E1 regions (PM-A/PM-B), according to the protocols previously validated for the detection of mucosal and cutaneous HPV genotypes. PCR products underwent sequencing analysis and the sequences were aligned to reference genomes from the Papillomavirus Episteme database. Phylogenetic analysis was then performed to assess the genetic relationships among the different sequences and between the sequences of the samples and those of the prototype strains. A broad spectrum of sequences related to mucosal and cutaneous HPV types was detected in 81% of the sewage samples analyzed. Surprisingly, sequences related to the anogenital HPV6 and 11 were detected in 19% of the samples, and sequences related to the “high risk” oncogenic HPV16 were identified in two samples. Sequences related to HPV9, HPV20, HPV25, HPV76, HPV80, HPV104, HPV110, HPV111, HPV120 and HPV145 beta Papillomaviruses were detected in 76% of the samples. In addition, similarity searches and phylogenetic analysis of some sequences suggest that they could belong to putative new genotypes of the beta genus. In this study, for the first time, the presence of HPV viruses strongly related to human cancer is reported in sewage samples. Our data increases the knowledge of HPV genomic diversity and suggests that virological analysis of urban sewage can provide key information useful in supporting epidemiological studies.

## Introduction

Papillomaviridae (PV) is a family of small epitheliotropic viruses of approximately 50–60 nm, with circular double stranded DNA genome 7–8 kb long, detected in all vertebrates. This family contains 16 genera named with the letters of the Greek alphabet. Human Papillomavirus (HPV) strains are classified into 5 genera: *alpha* (α), *beta* (β), *gamma* (γ), *mu* (μ), and *nu* (ν). The HPV members of the α genus primarily infect oral and genital mucosal surfaces and external genitalia, while HPVs belonging to the β, γ, μ, and ν genera infect non-genital mucosa and skin. Papillomaviridae is a rapidly growing family of viruses. In fact, most of the sequences of new viruses from humans and other vertebrates, that are uploaded on to databases, belong to this family [1]. Among the 120 HPV genotypes detected so far in the α genus, 30 infect anogenital epithelia and are the cause of sexually transmitted diseases (STDs). Of these, 15 have oncogenic potential and are called high-risk (HR). Women and men involved in the transmission of HPVs can be both asymptomatic vectors and victims of these infections. The HR genotypes HPV16, 18, 31, 33, 35, 39, 45, 51, 52, 56, 58, and 59 have been recognized as causal agents of cervical cancer (CC), the second most common cancer among women worldwide [2]. The genotype HPV16 is detected in 61% of CC clinical samples [4]. HPV16 and 18 have also been found to cause vaginal, vulval, anal and penile cancers. Moreover, half of oro-pharyngeal cancers are linked to HPV16 [2], [3]. Seven genotypes, HPV26, 53, 66, 67, 70, 73, and 82, could also be considered as probable carcinogenic candidates, while HPV6, 11, 40, 42, 43, 44, 54, 61, 72 and 81, causing anogenital warts, are considered low risk genotypes (LR-HPVs) [4]. It is worthwhile to note that genital warts represent a heavy burden among the female population; these symptoms are usually the impetus for the initial presentation by patients in consulting gynecologists. HPV6 and 11 are the most common genotypes detected in oro-pharyngeal cancer after HPV16, suggesting that these anogenital LR HPVs may indeed be malignant for the oral mucosa [5].

Skin HPVs are ubiquitous viruses involved in a variety of skin pathologies [6] but are also detected at a high prevalence in the normal skin of healthy subjects [7], [8]. Skin warts are caused most frequently by HPV1, 63 (μ genus), HPV2, 3, 4, 7, 10, 27, 57, 66 (α genus), and HPV4, 60, 64, 65, 75–77 (γ genus) [4], [6]. The association between β-HPVs and skin cancer was first recognized in patients with epidermodysplasia verruciformis (EV), a rare genetic disease. The EV-HPVs (HPV5, 8, 12, 14, 15, 19–25, 28, 29, 36–38) have been linked to non-melanoma skin cancer (NMSC), a pathology particularly frequent in immunosuppressed patients such as HIV-infected individuals and people receiving organ transplantation. Although some genotypes (HPV5, 8 and 38) display transforming activities in several experimental models [9], [10], epidemiological studies do not yet support any single genotype causing skin cancer in the general population [4]. HPV colonization of healthy skin occurs very early in life, and increases with age, as well as the prevalence of β and γ HPV species [11]. Conversely, the acquisition of specific immunological competence increases for the HPVs linked to NMSC while it decreases for those causing skin warts, as monitored by serological studies [12], [13]. Cutaneous HPVs persist in hair follicles, suggesting these sites as possible reservoirs. In contrast to CC, where HPV DNA is always present [4], no HPV DNA genome, or only few copies of it, persist in skin cancers. Interestingly, precursor lesions of skin cancer such as actinic keratosis show high viral loads compatible with a carcinogenic role of β-HPVs in the early events of cancer development [11].

HPV infections occur due to a failure of the innate or adaptive immune system. Immunosuppressed and HIV-infected individuals have a high prevalence of HPV infections, considered as opportunistic infections in AIDS. These infections do not appear to be diminished by HAART in HIV-positive individuals [14].

WHO recognizes the importance of CC and other HPV-related diseases as global public-health problems and recommends adopting and implementing routine vaccination in national immunization programs. Recent results indicate that the elimination of the genotypes included in Gardasil (HPV16, 18, 6 and 11) or Cervarix (HPV16, 18) vaccines may alter the evolutionary trajectory of circulating viruses and promote the evolution of new genotypes [15]; therefore, identification of the genotypes circulating in the population is of great importance for the development of both preventive and therapeutic programs of the HPV-associated diseases.

In the past few years, several studies have demonstrated the benefit of environmental surveillance as an additional tool in determining the epidemiology of different viruses circulating in a given community [16]–[23]. Sewer systems collect pathogens excreted in a range of body fluids from a wide area into a central facility. Therefore, the monitoring of centralized wastewater allows the detection of natural, accidental, or intentional contamination events [24]. Moreover, untreated wastewater provides a rich matrix in which novel viruses could be identified at the same time as studying virus diversity.

Recently the presence of human Polyomaviruses (HPy) has been described in urban sewage systems, enabling the assessment of the excretion levels and the potential risks of waterborne transmission by these viruses [25]. Moreover, the exploration of viral diversity by deep sequencing nucleic acids obtained from raw sewage, allowed the identification of 234 known viruses, including the newly discovered HPV112 and the HPy6 [26]. Both these viruses are skin-related, suggesting that viruses of human skin as well as those of stools can find their way into sewage.

The objective of the present study was to investigate whether DNA sequences of HPVs can be recovered from urban wastewaters, as has already been reported for other non-enteric viruses, and to assess the occurrence and distribution of HPV genotypes.

## Materials and Methods

### Sewage samples, concentration and DNA extraction

Forty-two inflow grab samples were collected from March 2011 to October 2011 at 14 Wastewater Treatment Plants (WTPs) of 8 cities from north to south of Italy: Torino, Genova, Venezia, Bologna, Roma, Cagliari, Bari, and Palermo. All the necessary permits were obtained for the described field study. Sample collection was performed in collaboration with the Italian Regional Agencies for Environmental Protection and Prevention (ARPA). Upon arrival each sample was named by a Sample ID (1528 to 1771), a unique individual identifier. Twenty ml of each sample were purified with glycine/chloroform and 10 ml used for DNA extraction by the NucliSens miniMAG nucleic acid isolation kit (BioMerieux). DNA was then eluted in 100 µl buffer and stored in aliquots at −80°C until use. A PostgreSQL database was previously created to keep track of all the primers, PCR products and samples used; an internet connection to this database is available for registered users at https://cosmos.bio.uniroma1.it. A murine Norovirus was added to the samples as an internal control to both calculate viral recovery efficiency and check for potential inhibitors by quantitative and qualitative PCR (data not shown), in case of negative PCR results.

### PCR assays and sequencing

Samples were analyzed using different sets of previously described primers, listed in Table 1, designed to detect both mucosal and cutaneous PVs. Each of the four diagnostic PCR assays, here called Method A, B, C and D, was performed using a 2-step amplification to either increase sensitivity or mitigate the inhibitory effect of substances possibly present in the samples. PCR conditions were as previously described, with some modification.

**Table 1:** PCR and primers used in this study.

Primer ID	Primer name	Sequence (5′>3′)	PCR ID	Product length (bp)	References
1778-f	MY11	GCMCAGGGWCATAAYAATGG	738	449	[27]
1777-r	MY09	CGTCCMARRGGAWACTGATC			
1779-f	GP5+	TTTGTACTGTGGTAGATACTAC	739	138	[28]
1780-r	GP6+	GAAAAATAAACTGTAAATCATATTC			
1781-f	SKF1	GAGCAAAATTTCCAACAAAAGG	740	210–238	[30]
1782-r	SKR1	ATACCATAGAYCCACTRGG			
1783-f	SKF2	AAATATCCTGATTATTTRGGMATG			
1784-r	SKR2	AAACYATAGAGCCACTWGG			
1785-f	PM-A	ACTGACCAAAGCTGGAAATC	742	117	[31]
1786-r	PM-B	TCTTGCAGAGCATTGAAACG			
1787-f	FAP59	TAACWGTIGGICAYCCWTATT	743	478	[29]
1788-f	FAP64	CCWATATCWVHCATITCICCATC			

#### Method A

the MY11/MY09 [27] primers were used for the first cycle of amplification and GP5+/GP6+ [28] for the nested reaction. The amplicon of 138 bp maps in the L1 major capsid protein-coding region. We also performed a hemi-nested assay (GP5+/MY09 primers) obtaining a 406 bp fragment. For each PCR reaction, 3 µl of the extracted DNA and 22 pmol of each primer were used in a final mixture of 25 µl, with 12.5 µl of GoTaq Green Master Mix 2× (Promega). Two µl of this mixture were subjected to a second round of PCR with 40 cycles of amplification. Samples positive to this method are reported in Table 2.

**Table 2:** PCR and sequencing results (L1 region).

Sample ID	WTP site	Nested PCR	PaVE prototype strains	Nt identity	Hemi-nested PCR	PaVE prototype strains	Nt identity
1528	Torino	−			+	HPV120	98.9%
1530	Torino	+	HPV11	96.6%	+	HPV11	99.7%
1530a	Torino	+	HPV11	99.1			
1535	Bari	−			+	HPV120	100%
1539	Palermo	+	HPV6	100%	+	HPV6	99.2%
1542	Palermo	+	HPV11	98.3%	+	HPV11	99.7%
1543	Cagliari	+	HPV16	100%	−		
1545	Cagliari	+	HPV6	98.3%	+	HPV6	100%
1545a	Cagliari	+	HPV6	100%			
1546	Cagliari	−			+	HPV120	99.7%
1547	Cagliari	+	HPV6	98.3%	+	HPV6	99.5%
1549	Genova	+	HPV11	98.3%	+	HPV11	99.7%
1550	Genova	+	HPV6	97.4%	+	HPV6	99.2%
1552	Roma	−			+	HPV107	82.1%
1556	Genova	−			+	HPV16	100%
1565	Venezia	+	HPV6	98.3%	−		
1568	Roma	+	HPV25	99.1%	+	HPV120	88.3%,
1570	Roma	−			+	HPV120	88%

PCR was performed by Method A (L1 consensus region), using MY11/MY09 for the first cycle and either GP5+/GP6+ primers (nested reaction), or GP5+/My09 (hemi-nested reaction) for the second run. The samples positive at least to one assay are included in the table. It shows in columns from the left: the sample ID numbers and WTP sites (white panel), the nested PCR results (grey panel), the hemi-nested PCR results (dark grey panel). The table also reports the HPV genotypes more closely related to the query sequences, and the percentages of nucleotide (nt) identity toward these prototypes obtained by BLAST analysis. The letter (a) indicates the sequences obtained after cloning.

#### Method B

the FAP59 and FAP64 degenerate primers were designed to target the L1 major capsid protein-coding region [29]. The first cycle of PCR was performed as a touchdown PCR to reduce nonspecific amplification. Two µl of this mixture were subjected to a second PCR round with annealing at 50°C, using the same primers to further amplify the fragment of interest, 478 bp long.

#### Method C

two pairs of degenerate primers SKF1/SKR1 and SKF2/SKR2 were used in a multiplex PCR obtaining a 210–238 bp fragment of the L1 region. The assay is able to detect β, γ and μ HPVs causing the cutaneous warts. A touchdown PCR was used in the first cycle like in method B. Two µl of this mixture were subjected to a second round of PCR with annealing at 45°C [30].

#### Method D

the PM-A/PM-B primers designed to detect 25 β-PVs were used [31]. The PCR assay amplifies a 117 bp fragment of the E1 helicase-coding region. The samples positive to method D are listed in Table 3.

**Table 3:** PCR and sequencing results (E1 region).

Sample ID	WTP site	PaVE prototype strains	Nt identity	Unassigned sequences
1528	Torino	HPV145	94.9%	
1529	Torino	HPV9, HPV104, HPV113	90%	Unassigned (1)
1530	Torino	HPV9, HPV104, HPV113	90%	Unassigned (1)
1531	Bari	HPV9, HPV104, HPV113	90%	Unassigned (1)
1532a	Bari	HPV9, HPV104, HPV113	90%	Unassigned (1)
1532	Bari	HPV9, HPV104, HPV113	89.2%	Unassigned (2)
1533	Bari	HPV9, HPV104, HPV113	89.2%	Unassigned (3)
1534	Bari	HPV9, HPV104, HPV113	90%	Unassigned (1)
1535	Bari	HPV9, HPV104, HPV113	90%	Unassigned (1)
1537	Palermo	HPV104	99.1%	
1537a	Palermo	HPV20	100%	
1538	Palermo	HPV9, HPV104, HPV113	90%	Unassigned (1)
1539	Palermo	HPV9, HPV104, HPV113	90%	Unassigned (1)
1540	Palermo	HPV9, HPV104, HPV113	90%	Unassigned (1)
1542	Palermo	HPV9, HPV104, HPV113	90%	Unassigned (1)
1543	Cagliari	HPV9, HPV104, HPV113	89.2%	Unassigned (4)
1543a	Cagliari	HPV 9	98.3%	
1544	Cagliari	HPV9, HPV104, HPV113	90%	Unassigned (1)
1544a	Cagliari	HPV9, HPV104, HPV113	89.2%	Unassigned (5)
1545	Cagliari	HPV9, HPV104, HPV113	90%	Unassigned (1)
1546	Cagliari	HPV9, HPV104, HPV113	90%	Unassigned (1)
1547	Cagliari	HPV9, HPV104, HPV113	90%	Unassigned (1)
1548	Cagliari	HPV9, HPV104, HPV113	90%	Unassigned (1)
1549	Genova	HPV9, HPV104, HPV113	90%	Unassigned (1)
1549a	Genova	HPV111	97.4%	
1550	Genova	HPV9, HPV104, HPV113	90%	Unassigned (1)
1550a	Genova	HPV110	99.1%	
1551a	Genova	HPV9, HPV104, HPV113	89.2%	Unassigned (6)
1551b	Genova	HPV9	98.3%	
1551c	Genova	HPV9	94%	
1553a	Roma	HPV9, HPV104, HPV113	90%	Unassigned (1)
1553	Roma	HPV9, HPV104, HPV113	89.2%	Unassigned (7)
1554	Roma	HPV9, HPV104, HPV113	90%	Unassigned (1)
1555	Genova	HPV9, HPV104, HPV113	90%	Unassigned (1)
1556	Genova	HPV9, HPV104, HPV113	90%	Unassigned (1)
1557	Genova	HPV76	95.7%	
1561	Genova	HPV80	98.3	
1565	Venezia	HPV9, HPV104, HPV113	90%	Unassigned (1)
1566	Venezia	HPV9, HPV104, HPV113	90%	Unassigned (1)
1568	Roma	HPV9, HPV104, HPV113	90%	Unassigned (1)
1571	Roma	HPV9, HPV104, HPV113	90%	Unassigned (1)

PCR was performed by method D, using PM-A/PM-B primers (E1 consensus region). Only the positive samples are included in the table. In columns are shown from the left: sample ID numbers, sites of the WTP, the HPV genotypes more closely related to the query sequences, and the percentage of nucleotide (nt) identity toward these prototypes obtained by BLAST analysis. The sequences obtained from different clones are indicated by alphabetical code (ID sample “a”, “b”). Seven different sequences, showing less than 90% identity with more than one prototype, are indicated as unassigned sequences with progressive numbers (1–7) in brackets.

All standard precautions adhering to strict laboratory practices were followed to prevent any PCR contamination. PCR reactions were purified with Montage SEQ96 Sequencing Reaction Cleanup kit (Millipore Corporation) and labeled using ABI-PRISM BigDye Terminator Cycle Sequencing kit (Applied Biosystems). Unincorporated dye terminators were removed using a Montage SEQ96 Sequencing Reaction Cleanup Kit (Millipore Corporation). Sequencing analyses were performed in a capillary automatic sequencer (ABI-PRISM 310 Genetic Analyser, Applied Biosystems).

**Figure 1:**
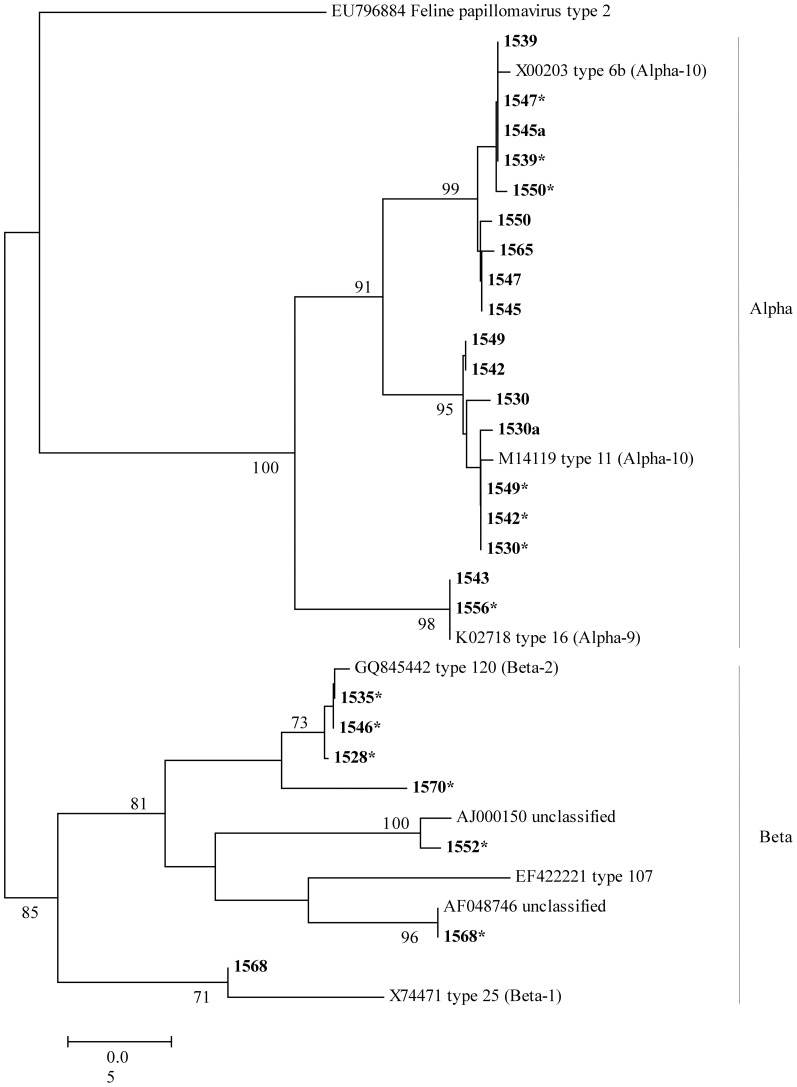
Phylogenetic tree constructed with the sequences obtained by Method A (L1 region). The tree was constructed using the Maximum Likelihood method based on the Kimura 2-parameter model. The sequences identified in this study are in bold. The percentage of trees in which the associated taxa clustered together is shown next to the branches. Bootstrap values higher than 70% are shown. HPV prototypes reported in the phylogenetic tree are: HPV6b, X00203; HPV11, M14119; HPV16, K02718; HPV25, X74471; HPV120, GQ845442; AJ000150; AF048746. A feline papillomavirus type 2 (EU796884) was included as an outgroup. Identical sequences obtained from the different plasmid cloned or identical sequences obtained from the nested and hemi-nested amplicons were not included in the tree. Sequences obtained from different clones were given an alphabetical code (ID sample “a”, “b”); sequences obtained from the hemi-nested reaction were marked with an asterisk.

**Figure 2:**
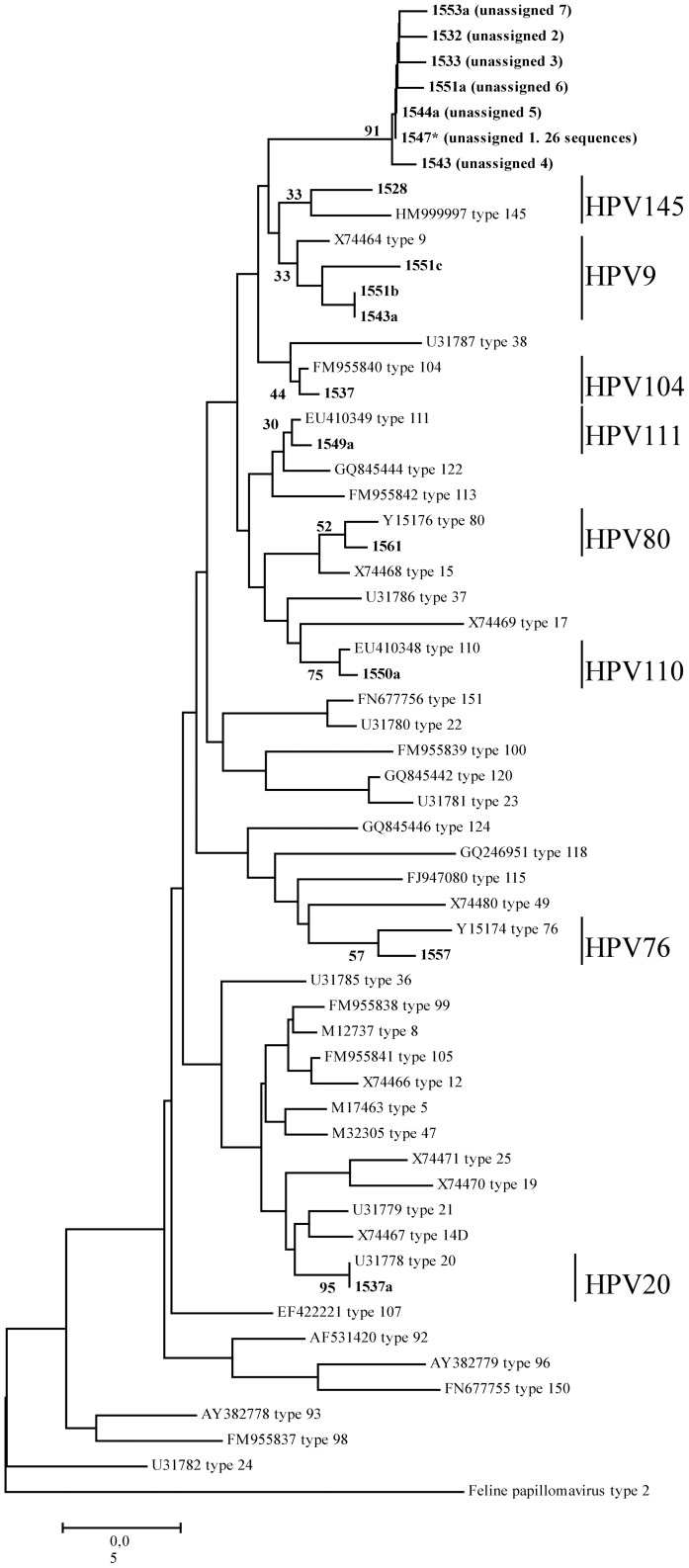
Phylogenetic tree constructed with the sequences obtained by Method D (E1 region). The tree was constructed using the Maximum Likelihood method based on the Kimura 2-parameter model. The sequences identified in this study are in bold. The percentage of trees in which the associated taxa clustered together is shown next to the branches. Only selected bootstrap values related to the studied sequences are displayed. The Beta HPV prototypes reported in the phylogenetic tree are: HM999997 type 145, X74464 type 9, U31787 type 38, FM955840 type 104, EU410349 type 111, GQ845444 type 122, FM955842 type 113, Y15176 type 80, X74468 type 15, U31786 type 37, X74469 type 17, EU410348 type 110, FN677756 type 151, U31780 type 22, FM955839 type 100, GQ845442 type 120, U31781 type 23, GQ845446 type 124, GQ246951 type 118, FJ947080 type 115, X74480 type 49, Y15174 type 76, U31785 type 36, FM955838 type 99, M12737 type 8, FM955841 type 105, X74466 type 12, M17463 type 5, M32305 type 47, X74471 type 25, X74470 type 19, U31779 type 21, X74467 type 14D, U31778 type 20, EF422221 type 107, AF531420 type 92, AY382779 type 96, FN677755 type 150, AY382778 type 93, FM955837 type 98, and U31782 type 24. A feline papillomavirus type 2 (EU796884) was included as an outgroup. Sample 1547, marked with* had identical sequences with other 25 samples (IDs 1529, 1530, 1531, 1532a, 1534, 1535, 1538, 1539, 1540, 1542, 1544, 1545, 1546, 1548, 1549, 1550a, 1553, 1554, 1554a, 1555, 1556, 1568, 1566, 1571), not included in the tree. Sequences obtained from different clones are indicated by alphabetical code (ID sample “a”, “b”).

In order to identify the PV genotypes, the PCR products of the expected size were both directly sequenced and cloned into the pGEM-T vector (Promega). Recombinant DNA plasmids were extracted by FastPlasmid Mini kit (Eppendorf) and sequenced (3 clones per sample) by vector-specific primers. The sequences obtained from different clones are marked with an alphabetical code (ID sample “a”, “b”) in Tables 2 and 3 and in Figures 1 and 2.

### Genotyping and phylogenetic analysis

The sequences obtained from PCR amplicons were aligned to prototype sequences of the Papillomavirus Episteme database (PaVE) (http://pave.niaid.nih.gov/#home) to find the most related reference genotypes, and as a control, to sequences of the NCBI GeneBank database (http://blast.ncbi.nlm.nih.gov/Blast.cgi), using the Basic Local Alignment Search Tool (BLAST).

Phylogenetic analysis was performed using MEGA software version 4.0 [32]. Nucleotide sequences were aligned using the Clustal W algorithm. The phylogenetic tree was constructed using the Maximum Likelihood method based on the Kimura 2-parameter model, integrated into the MEGA software. The robustness of the clustering results was assessed by bootstrap resampling (1000 replicates). Consensus sequences were submitted to the EMBL Nucleotide Sequence Database using the Webin Submission Tool available at http://www.ebi.ac.uk/embl/Submission/.

## Results

Inflow grab samples were analyzed by PCR using four PV-specific molecular methods employing the MY09/MY11 and GP5/GP6 (Method A), FAP59/64 (Method B), SKF/SKR (Method C), and PM-A/PM-B (Method D) set of primers as described in Materials and Methods. Nucleic acid extraction efficiency, calculated on randomly selected samples, showed an average recovery exceeding 35%. PCR inhibitors were not detected in the negative samples. The amplicons were both directly sequenced and sequenced after cloning. In order to identify the more closely related PV genotypes, the sequences were submitted to BLAST analysis and compared to both the interactive Papillomavirus Episteme database (PaVE), a database for the Papillomaviridae family containing 241 reference complete genomes, and to the NCBI GenBank database.

Only methods A and D gave positive results, summarized in Tables 2 and 3, which report sample ID numbers, sites of the WTPs, PCR results, the more closely related prototypes and percentage of nucleotide (nt) identity. Eighty-one percent of the samples (34/42) were HPV-positive using method A or D, or both. PCR assays using methods C and B resulted in multiple, non-specific amplification products. In Table 2, thirty-eight % (16/42) of the samples were positive by method A. Three sequences related to the α-types HPV6, 11, and 16, and 2 sequences related to the β-types, HPV25 and 120, showing more than 97% of nucleotide (nt) identity with the PAVE sequence database, were identified by sequence analysis. In some cases, different results were obtained from the same sewage sample by either GP5+/GP6+ in nested or GP5+/MY09 in hemi-nested PCR assays (Table 2). Out of the 16 positive samples, 9 were positive by both the assays, 6 were positive only by hemi-nested and one was positive by nested PCR. The 406 bp amplicons detected by hemi-nested PCR in the samples ID 1552, 1568 and 1570, showed less than 89% of nt identity with prototypes of the PAVE database (see Table 2), related to HPV107 and HPV120. The comparison of these sequences with the GenBank database, showed greater matches for samples 1552 and 1568: the first showed 96% nt identity with a HPV partial sequence, previously detected in an esophageal carcinoma sample from China, submitted as an unclassified sequence (AJ000150); the second showed 100% of nt identity with an unclassified sequence of a human clinical oral sample from USA (AF048746), thus making it impossible to assign these sequences to known genotypes. As for sample ID 1570, similarity search against PaVE or GenBank gave an identical result which was of 88% nt identity with the reference strain HPV120 (see Table 2). Since the L1 sequences of the 1552, 1568, and 1570 samples differ by more than 10% with prototype genomes, we speculate that they may belong to putative new genotypes [1], [33].

Different results were obtained by sequencing the nested and hemi-nested products. In one case (sample ID 1568, Table 2), a sequence related to HPV25 and a putative novel genotype was detected by nested and hemi-nested assay, respectively. In samples 1530 and 1545 (Table 2, nested PCR results) different sequences belonging to the same genotype were detected by direct sequencing or sequencing after cloning of the PCR products. These results suggest the co-presence of several strains in a single sewage sample.

The results of the phylogenetic study, performed on the sequences obtained by method A (L1 region), are presented in Figure 1. The tree includes the sequences obtained in this study, in addition to the prototype sequences from the PaVE database and from GeneBank (see figure legend). Sequences from sewage samples are grouped into two different clusters corresponding to alpha and beta HPVs, according to the genotyping results by BLAST analysis. The alpha cluster is subdivided into two main groups, referred to as α9 (HPV16, 98% bootstrap) and α10 (HPV6 and 11, 91% bootstrap) species. Sewage samples form well-supported groups with their corresponding prototype strains: HPV6 (ID 1539, 1545, 1547, 1550 and 1565), HPV11 (ID 1530, 1542 and 1549), and HPV16 (ID 1543 and 1556). In the β cluster, samples 1528, 1546 and 1535 are grouped with the prototype HPV120 (73% bootstrap); sample 1568 clusters with HPV25 (71% bootstrap). The HPV sequences detected by hemi-nested PCR in ID 1552 and 1568, showing percentages of nt identity lower than 89% with the HPV107 and HPV120 prototypes from PaVE databank, cannot be clustered in supported groups with these genotypes, but are grouped with the unclassified partial sequences AJ000150, and AF048746 from GeneBank, mentioned above (bootstrap 100% and 96% respectively). Not even the partial L1 sequence of sample ID 1570 can be placed with certainty in a definite cluster. This leads us to believe that in all these cases the phylogenetic analysis supports the hypothesis that these sequences could belong to putative new HPV genotypes.

A high percentage of sewage samples (76%) tested positive by the Method D, designed for the detection of β-HPV DNA [31]. The results are reported in Table 3. Sequence analysis allowed us to detect different HPV strains by cloning and sequencing of PCR products in samples ID 1530, 1537, 1543, 1544, 1549, 1550, 1553 (2 strains) and 1551 (3 strains, see Table 3). The sequences obtained from different clones are indicated by alphabetical code (ID sample “a”, “b”) in Table 3 and Figure 2. BLAST analysis results showed that only a small number of amplicons had a nt identity higher than 94% with the prototypes from PaVE database. These sequences were related to HPV9 (ID 1543a, 1551b and 1551c), HPV20 (ID 1537a), HPV76 (ID 1557), HPV80 (ID 1561), HPV104 (ID 1537), HPV110 (ID 1550a), HPV111 (ID 1549a) and HPV145 (ID 1528). Thirty-two amplified sequences showed a nt identity equal to or less than 90% with the HPV9, HPV104, and HPV113 prototypes (Table 3), thus remaining “unassigned” to a specific genotype. Of these, 26 sequences were identical (Table 3, unassigned-1) while 6 were unique (Table 3, unassigned 2 to 6).

The results of the phylogenetic study performed with the sequences obtained by Method D, are presented in Figure 2. All β-HPV prototype sequences from the PaVE database were included into the tree to make an accurate comparison (see figure legend). The prototype sequences segregate into separated branches of the tree, thus indicating that partial sequencing of the E1 region permits to distinguishing among different genotypes. All sequences showing more than 94% of nt identity with the PaVE prototypes can be clustered with the corresponding reference genomes, confirming the results obtained by BLAST analysis, even if the bootstrap values in several branches are low (see Figure 2). Of note, the 32 sequences sharing a percentage of nt identity lower than 90% with HPV9, HPV104, and HPV113 (unassigned 1–7, Table 3), formed a well-supported group (91% bootstrap) but did not cluster with any of these prototypes (Figure 2, top). The genetic variability within the unassigned sequences, assessed by computation of pairwise distances, was 0.016 (number of base substitutions per site), attesting that these sequences may belong to the same genotype, possibly a not yet discovered β-PV.

The sequences detected in this study have been submitted to GeneBank with the following accession numbers: HE80564 to HE805665.

## Discussion

The objective of the present study was to investigate the occurrence and genetic diversity of HPVs in urban wastewaters through molecular screening of raw sewage samples. The results showed the presence of sequences related to α-HPVs (HPV6, 11, 16) and β-HPVs (HPV9, 20, 25, 76, 80, 104, 110, 111, 120, and 145). In our study, human Papillomavirus belonging to γ, μ or ν genera were not detected.

The different assays used allowed us to establish that multiple virus genotypes and multiple virus strains belonging to the same genotype (up to three different sequences) were present in a single sewage sample, confirming that urban wastewaters represent a rich matrix for studying viral diversity.

Our data show that the MY11/MY09 and GP5+/GP6+ primer sets, mapping in the major capsid L1 protein (method A) and validated on clinical samples, are powerful tools for HPV detection even in sewage samples. The use of hemi-nested primers in addition to nested primers, allowed us to increase the number of genotypes detectable and to detect a higher percentage of positive samples, in agreement with data obtained by other authors [34]. Surprisingly, the low risk HPV6 and 11 were the most prevalent α genotypes detected in sewage samples. The high risk HPV16 genotype was detected in two samples. It is noteworthy that HPV16, 6 and 11 are the genotypes most frequently detected in cancers of the oral cavity [2], [4], [5], [11]. In addition to viruses of the α genus, the L1-primers were also able to detect viruses belonging to the β-group species such as HPV25 (β1) and HPV120 (β2). The 405 bp sequences found in the ID samples 1552, 1568 and 1570 showed a percentage of identity lower than 88% with HPV107 and HPV120 prototypes of PaVE database and could not be clustered in supported groups with these reference genomes in the phylogenetic tree. On the basis of the results obtained by BLAST and phylogenetic analysis, these sequences could belong to putative novel genotypes. In fact, according to the guidelines for PV nomenclature (Study Group of Papillomaviruses of the International Committee on Taxonomy of Viruses), a PV genotype species differs in the L1 gene sequence by at least 10% from the genes of other known genotypes [1], [33]. However, in order to be officially recognized as a unique genotype, the complete genome must be sequenced and deposited in the form of clones to the Reference Centre for Papillomaviruses in Heidelberg, Germany [1], [33]. However, sequencing of the full genome will not be an easy task due to the complexity of the urban wastewater matrix. Putative new HPV genotypes are continuously being discovered within the *Papillomaviridae* family, and an even larger number is expected to exist. Therefore a great effort is required to characterize these viruses and elucidate their implication in human health.

Method B and C, based on FAP and SK couples of primers, did not give any positive result. In fact, the PCR amplification products consisted of a mixture of fragments differing in length that hampered the identification of HPV amplicons of the expected size. These tests use degenerated primers which, in a complex matrix such as wastewater, may amplify non-specific products. Wastewater indeed represents the most challenging matrix for molecular identification of pathogens, due to the presence of a spectrum of microorganisms, cell debris, proteins, lipids, and various potential inhibitors.

The PCR assay performed on the E1 region by PM-A/PM-B primers showed a high sensitivity in detecting β-HPV sequences in sewages. These primers were able to detect viruses of the β1 (HPV20), β2 (HPV9, 80, 104, 110, 111, and 145) and β3 (HPV76) species [1].

The phylogenetic tree constructed in the E1 region allowed us to cluster some sequences with the reference genotypes with a high degree of reliability, confirming BLAST results, even if the bootstrap values in several branches were low. The E1 region targeted by the PM-A/PM-B primers codes for the helicase gene, involved in viral replication. Since this region is highly conserved in PVs, it has a lower discrimination power compared to the major capsid protein L1, which is indeed the region of choice for HPV genotyping and classification.

On the other hand, the PM-A/PM-B primers which were originally designed to amplify a variable part of the E1 region, were able to discriminate 25 human papillomavirus genotypes of the beta-papillomavirus genus (5, 8, 9, 12, 14, 15, 17, 19, 20, 21, 22, 23, 24, 25, 36, 37, 38, 47, 49, 75, 76, 80, 92, 93 and 96 [31]. Here we demonstrate that this primer pair has an even broader range of action, being able to detect also the recently classified beta HPV 104, 110, 111 and HPV145 [1].

The majority (78%, 32 sequences) of E1 amplicons obtained by Method D, were not assigned to any HPV genotype by both sequence similarity searches and phylogenetic analysis. These unassigned sequences showed a very low genetic diversity from each other, indicating that they may belong to the same genotype, still unknown. This virus could represent a beta genotype ubiquitously present on human skin, which explains its presence in the vast majority of our WTPs.

The unexpectedly high HPV DNA prevalence in raw sewage samples probably reflects a high diffusion of HPVs in the human population. Cutaneous and mucosal viruses can find their way into sewage through both healthy and torn skin, and washing of mucous secretions. It is well known that the treatment procedures cannot completely eliminate viruses present in wastewater [19], [35], [36], thus permitting their spread in the environment. However, the presence of HPV-DNAs in sewage samples does not necessarily imply the infectivity of these samples. Unfortunately, the absence of conventional *in vitro* systems able to support the HPV replication prevents the study of infectivity for any HPV isolated from any source. Since DNA viruses show a high resistance to inactivating treatments and a slow loss of infectivity [37], we cannot exclude the possibility that infectious HPVs spread in the environment even after wastewater treatments and cause infections through contact with skin or mucosa. The HPV16, 6 and 11 are known causes of sexually transmitted infections; however, current evidences suggest that these HPVs can also be transmitted non-sexually. In fact, cases of vertical and horizontal transmission of HPVs and autoinoculation through contact of the body have been clearly documented for HPV16, 6 and 11. Therefore, the HPV presence in environmental samples opens up the possibility of a fomite or waterborne transmission. To our knowledge, no evidence has been reported that contaminated toilet seats, doorknobs, towels, soaps, swimming pools, or recreational waters, can transmit anogenital HPVs. Nevertheless, these modalities of infection could explain some cases of anogenital HPV transmission in children, virgins and nuns, none of them being involved in sex-abuse or sexual intercourse [4], [38]. Certainly, understanding the implications in human health of the presence in the environment of oncogenic viruses including HPVs, and the potential viral waterborne transmission are great challenges for future studies [39].

Regarding the genotypes detected in our study, the presence of HPV16, 6 and 11 in sewage samples probably reflect the high pathogenicity of these viruses. On the other hand, the epidemiology of the β-HPVs is less known. Several genotypes detected in our study are EV-HPVs (HPV9, 20, 25, 80) that have been recently linked to precursor lesions of skin cancer with HPV110, recently classified [40]. We noticed that, with the exclusion of HPV76, the β-HPVs found in our study have been detected also in mucosal tissues; consequently, the majority of the HPVs found in wastewaters were of mucosal origin [4], [41]. We were not able to determine whether this was due to screening a relatively small number of samples or whether it reflected higher viral load and greater resistance of mucosal HPVs compared to cutaneous HPVs, in wastewaters.

The present study is the first report on the occurrence of HPV viruses, strongly related to human cancer, in sewage samples, and increases our knowledge on the HPV genomic diversity.

In conclusion, our results suggest that the surveillance of sewer systems, successfully employed for monitoring of enteric viruses, could be also applied to epitheliotropic viruses such as the oncogenic HPVs. Although studies on a larger-scale are required to obtain a well-defined picture of the HPVs present in environmental samples, our study contributes significantly to this effort. The environmental data, integrated and compared to published clinical data, can be used in statewide surveillance programs to monitor prevalent, novel and emerging genotypes circulating in the community and to assess any discrepancies with genotypes included in the current vaccines.
